# Diet-Dependent Effects of Intracerebroventricular Maresin-1 on Hypothalamus of Rats

**DOI:** 10.3390/ijms27188039

**Published:** 2026-09-09

**Authors:** Cihan Suleyman Erdogan, Irem Solmaz, Cansu Yakin, Fatma Ozge Tuncel, Tuba Keskin, Suat Tekin, Suleyman Sandal, Bayram Yilmaz

**Affiliations:** 1 Department of Physiology, Faculty of Medicine, Yeditepe University, Istanbul 34755, Türkiye; cihan.erdogan@yeditepe.edu.tr (C.S.E.); cansu.yakin@yeditepe.edu.tr (C.Y.); fatmaozge.tuncel@std.yeditepe.edu.tr (F.O.T.); 2 Department of Physiology, Faculty of Medicine, Inonu University, Malatya 44280, Türkiye; iremslmzz.0@gmail.com (I.S.); keskintuba1992@gmail.com (T.K.); suat.tekin@inonu.edu.tr (S.T.); 3 Department of Physiology, Faculty of Medicine, Dokuz Eylul University, Izmir 35210, Türkiye; 4 Izmir Biomedicine and Genome Center, Izmir 35340, Türkiye

**Keywords:** AgRP, diet-induced obesity, metabolic hormones, Maresin-1, POMC

## Abstract

Obesity is associated with hypothalamic dysfunction, including altered arcuate nucleus (ARC) signaling-related impairment of energy homeostasis. However, the central metabolic effects of the specialized pro-resolving mediator Maresin-1 (Mar1) remain unclear. This study investigated whether central Mar1 administration alters ARC agouti-related peptide (AgRP), proopiomelanocortin (POMC), and metabolic hormone profiles in rats under standard diet (STD) and high-fat diet (HFD) conditions. Male Wistar albino rats were fed either an STD or an HFD for 12 weeks and assigned to untreated control, vehicle, 50 ng/kg/day Mar1, or 100 ng/kg/day Mar1 groups within each diet condition. Mar1 was administered by continuous intracerebroventricular infusion for 7 days. Body weight, food intake, serum insulin, leptin, ghrelin levels, and ARC AgRP and POMC expressions were evaluated. Central Mar1 infusion did not alter body weight or food intake. In STD-fed rats, 100 ng Mar1 significantly increased serum insulin compared to control (*p* = 0.025), both Mar1 doses significantly increased leptin versus control (*p* = 0.0003 and *p* = 0.0091, respectively), and 50 ng Mar1 significantly increased ghrelin compared with control and vehicle groups (*p* = 0.0016 and *p* = 0.0055, respectively). In HFD-fed rats, Mar1 did not significantly change serum hormone levels, and the difference in ghrelin between the 50 ng Mar1 and vehicle groups did not reach statistical significance (*p* = 0.051). Mar1 did not alter AgRP expression in the ARC under either diet condition. However, under HFD, 100 ng Mar1 increased POMC expression relative to control, vehicle, and 50 ng Mar1 groups (*p* = 0.0076, *p* = 0.0078 and *p* = 0.0204, respectively). Central Mar1 infusion therefore altered circulating metabolic hormones with no significant diet x treatment interaction, and increased ARC POMC expression under HFD, an effect supported by a significant diet x treatment interaction (*p* = 0.0091), without changing energy intake or body weight over the 7-day infusion. These findings indicate a diet-dependent modulation of hypothalamic melanocortin tone rather than a significant effect on energy balance.

## 1. Introduction

Obesity is characterized by excessive fat accumulation and is associated with various chronic non-communicable diseases, including type 2 diabetes (T2D), metabolic syndrome, cardiovascular diseases and cancer [1,2]. It is a global public health problem and has emerged as an epidemic affecting individuals regardless of demographics [3,4], and it is suggested that more than 60% of adults older than 25 years will be overweight or obese by 2050 [3]. The major factor leading to obesity is deregulated calorie utilization and positive energy balance due to various factors including genetic predisposition, exposure to environmental factors, overnutrition, excess high-fat diet (HFD) consumption, sedentary lifestyle and socio-economic status [5,6,7,8]. This chronic energy surplus promotes adipose tissue expansion and low-grade inflammation, which, in turn, precipitates systemic metabolic disturbances [9,10]. Consequently, obesity-associated metabolic dysregulation is accompanied by endocrine and neural adaptations that progressively impair the ability of the organism to maintain energy homeostasis [11].

Energy homeostasis is tightly regulated by the neural circuits located in the brain, with the hypothalamus serving as a central integrative hub for circulating metabolic signals and downstream control of food intake, energy expenditure and glucose homeostasis [12,13,14,15,16,17]. A central node in the regulation of homeostatic appetite is the arcuate nucleus of the hypothalamus (ARC), containing interconnected neural populations exerting opposing effects on feeding that are highly sensitive to peripheral energy signals [11,18,19]. The best-characterized neuronal populations in the ARC regulating homeostatic food intake are agouti-related peptide (AgRP) and proopiomelanocortin (POMC) neurons [20,21,22]. AgRP neurons co-express neuropeptide Y (NPY) and produce orexigenic peptides and stimulate food intake, whereas POMC neurons produce α-melanocyte-stimulating hormone (α-MSH) to suppress appetite [22]. The activity of these neuronal populations is shaped by peripheral hormones such as leptin, insulin, and ghrelin, placing the ARC at the center of neuroendocrine regulation of appetite and body weight [11,16,23,24].

Diet composition critically influences both onset and the phenotype of obesity [25,26]. HFD paradigms are widely used to model diet-induced obesity (DIO) because increased dietary energy density and palatability promote hyperphagia and accelerate weight gain, while also recapitulating key metabolic features of human obesity [26,27,28]. In addition to its effects on adiposity and endocrine profiles, HFD feeding is widely associated with the development of chronic low-grade inflammation in metabolic tissues, particularly adipose tissue, which contributes to systemic metabolic dysfunction [29]. Beyond peripheral tissues, HFD consumption has also been shown to activate pro-inflammatory signaling in the hypothalamus, impair anorexigenic insulin signaling within the hypothalamus, and induce hypothalamic stress and gliosis, indicating that DIO involves central inflammatory and stress-related processes in brain regions essential for body weight regulation [30,31,32].

Specialized pro-resolving mediators (SPMs), including resolvins, protectins, and maresins, are enzymatically derived from polyunsaturated fatty acids and act through specific receptors to terminate inflammation, enhance efferocytosis, and support tissue repair without immunosuppression [33]. Impaired SPM biosynthesis and/or actions contribute to persistent low-grade inflammation and metabolic dysfunction, thereby sustaining insulin resistance and tissue pathology [34,35,36]. Maresin-1 (Mar1) is a docosahexaenoic acid (DHA)-derived SPM originally identified in macrophage-mediated resolution responses and is increasingly recognized as a regulator of immunometabolic homeostasis [37,38]. Mechanistically, Mar1 has been shown to signal via activation of the leucine-rich repeat-containing G-protein-coupled receptor 6 (LGR6) [37] and retinoic acid-related orphan receptor alpha (RORα)/12-lipoxygenase to promote inflammation resolution [39]. Previous experimental studies reported that Mar1 exerts beneficial metabolic activities in obesity [40] and hepatic steatosis in rodents [41,42]. In addition, although human data remain comparatively limited, reduced circulating Mar1 has been reported in metabolic disease states such as T2D [43]. Collectively, these findings indicate that Mar1 modulates peripheral metabolic and inflammatory pathways in rodent models of obesity and hepatic steatosis, and that its circulating levels are reduced in human metabolic disease. Whether these observations translate into therapeutic efficacy in obesity has not been established, and the central component of Mar1 action in particular remains largely unexplored.

Despite evidence for peripheral metabolic benefits, the extent to which Mar1 modulates central appetite-regulating circuits, particularly ARC AgRP and POMC neuropeptide levels, remains unclear. As HFD exposure can induce neuroendocrine resistance states that promote hyperphagia, investigating whether Mar1 can normalize appetite-related neuropeptide signaling and key metabolic hormones is directly relevant. Accordingly, the aim of the present study was to investigate whether central Mar1 infusion modulates levels of hypothalamic neuropeptides, AgRP and POMC, alongside circulating leptin, ghrelin, and insulin concentrations in male rats fed with a standard diet (STD) or an HFD, and to clarify a potential brain–periphery mechanism through which Mar1 may act.

## 2. Results

The two dietary groups were indistinguishable at the start of the study (55.2 ± 4.0 g under STD versus 55.2 ± 3.7 g under HFD, *p* > 0.99), confirming successful randomization. By the end of the 12-week feeding period, before any treatment was given, HFD-fed rats were substantially heavier than STD-fed rats (486.2 ± 34.8 g versus 422.6 ± 29.3 g, *p* < 0.0001) and had gained 17.3% more weight over the feeding period (431.0 ± 33.6 g vs. 367.5 ± 28.4 g, *p* < 0.0001). Naso-anal length was also greater under HFD (24.6 ± 1.0 cm versus 23.8 ± 1.0 cm, *p* = 0.003). Because the Lee index is the ratio of the cube root of body weight to naso-anal length, this parallel increase in length meant that the index did not differ between diets (0.322 ± 0.013 versus 0.317 ± 0.015, *p* = 0.14). It is important to note that the Lee index was not used as a primary criterion for obesity confirmation in this study. As previously reported by Stephens [44] and Bernardis [45], the Lee index correlates poorly with actual carcass fat in adult rats of similar age and nutritional history, largely because naso-anal length is an unreliable predictor of fat-free mass under these conditions. Consistent with this limitation, 29 of 32 STD-fed animals also exceeded the conventional 0.3 threshold, further demonstrating the index’s poor discriminatory power in our experimental setting.

**Figure 1:**
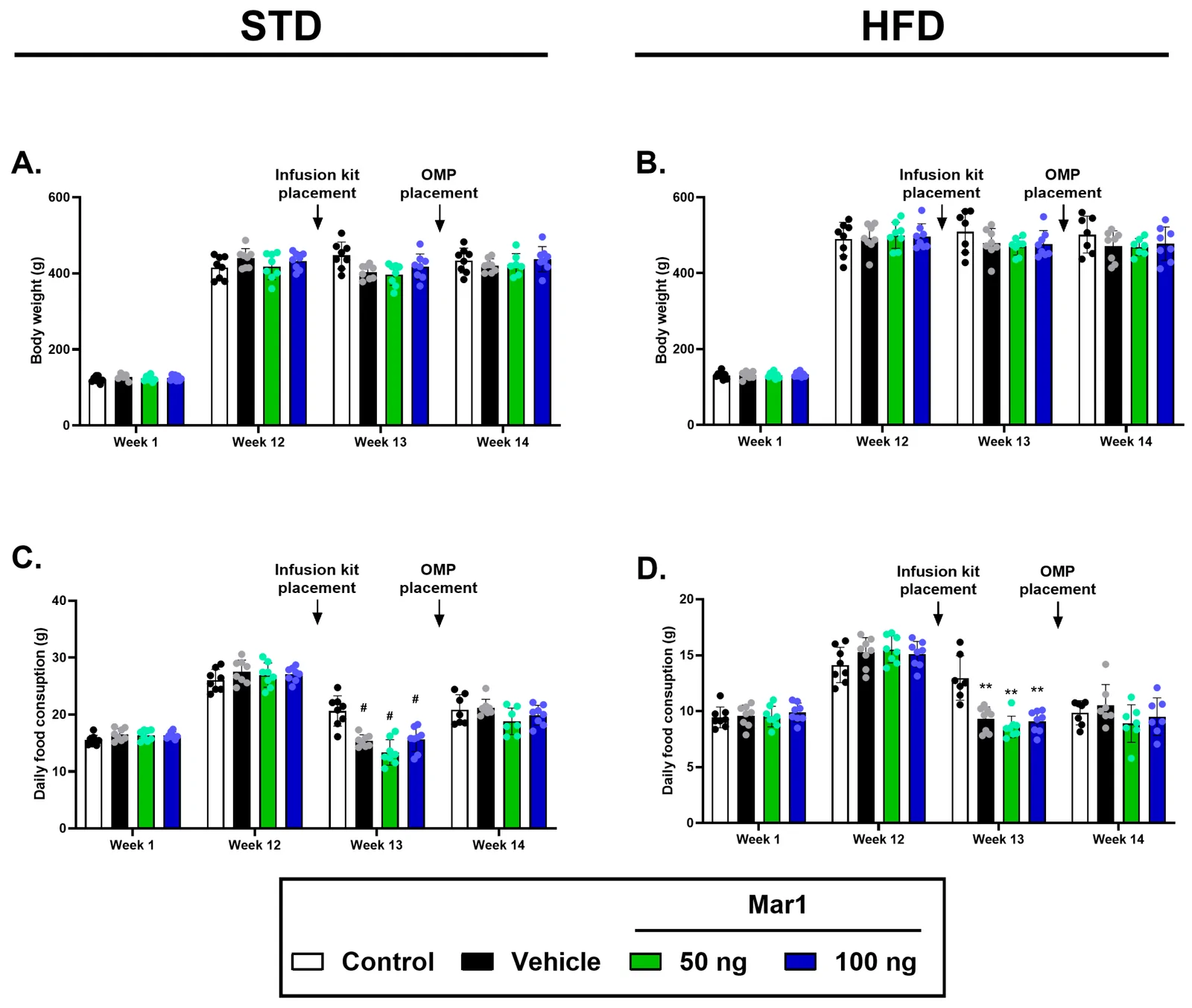
Effects of Mar1 infusion on body weight and food consumption in rats fed an STD or an HFD. (**A**,**B**) Body weight and (**C**,**D**) daily food consumption were monitored for 14 weeks. Arrows denote infusion kit placement and OMP placement. (Statistical analysis: Two-way ANOVA followed by Tukey’s multiple comparison test. ** *p* < 0.01 and ^#^ *p* < 0.0001 vs. the respective control groups).

One week of Mar1 infusion did not alter the body weight of the rats in either feeding regimen, the main effect of treatment being non-significant under both diets (Figure 1A,B, for STD, F(3, 111) = 1.915, *p* = 0.1312 and for HFD, F(3, 109) = 1.345, *p* = 0.2636). Daily food consumption in STD-fed rats was similar among groups at baseline and at week 12 (Figure 1C). However, a transient reduction in food intake was observed at week 13 in all groups that underwent surgical procedures (vehicle and both Mar1-treated groups), reflecting the expected postoperative stress response (F_Interaction_(9, 110) = 7.287, *p* < 0.0001 for the week x treatment interaction; control vs. vehicle, 50 ng and 100 ng at week 13, all *p* < 0.0001), while the untreated control group, which did not undergo surgery, maintained stable food intake (Figure 1C). By week 14, food consumption was comparable across all groups (Figure 1C). A similar pattern was observed in HFD-fed rats, with a transient postoperative decline in food intake at week 13 in the vehicle and Mar1-treated groups compared to the untreated control group (F_Interaction_(9, 79) = 8.036, *p* < 0.0001, for the week x treatment interaction in the mixed-effects analysis; control vs. vehicle, 50 ng and 100 ng at week 13, both *p* < 0.0001), followed by recovery to control levels by week 14 (Figure 1D).

**Figure 2:**
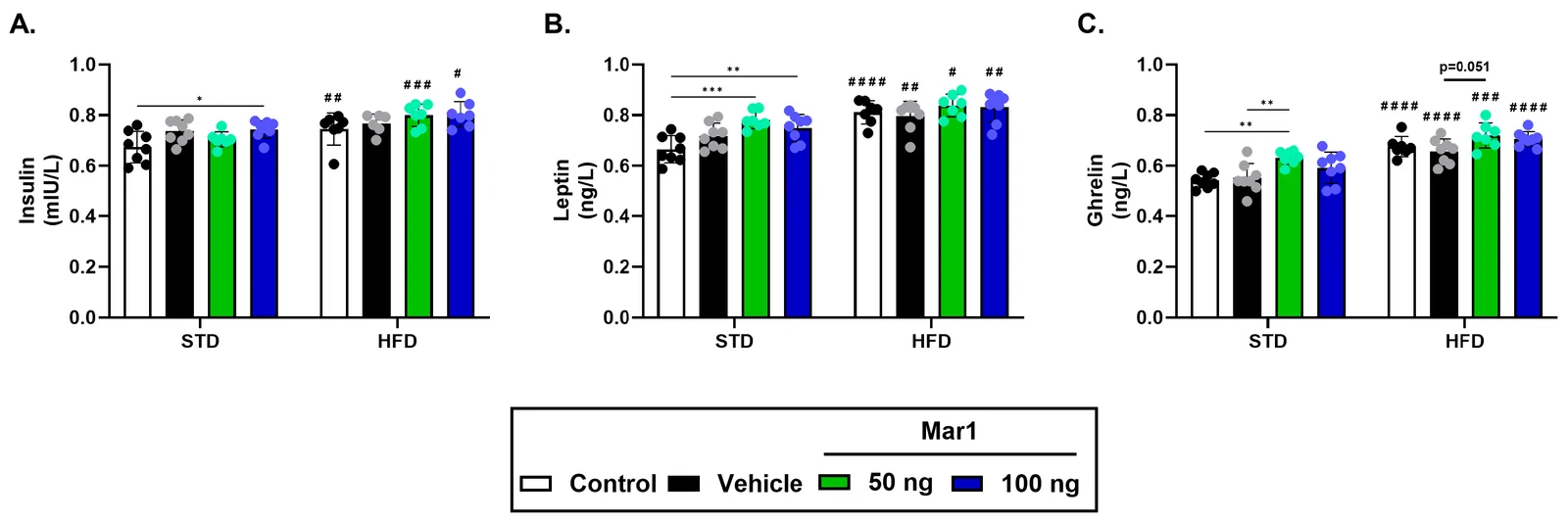
Effect of Mar1 infusion on serum (**A**) insulin, (**B**) leptin and (**C**) ghrelin levels of the animals fed either an STD or an HFD. (Statistical analysis: Two-way ANOVA followed by Tukey’s multiple comparison test. Asterisks denote comparisons between treatment groups within a diet (* *p* < 0.05, ** *p* < 0.01, *** *p* < 0.001); hash symbols denote comparison of the same treatment group between diets (^#^ *p* < 0.05, ^##^ *p* < 0.01, ^###^ *p* < 0.001 and ^####^ *p* < 0.0001 vs. respective treatment-matched STD-fed group).

For all three hormones, diet produced a significant main effect and treatment produced a smaller but significant main effect, whereas the diet x treatment interaction was not significant in any case (insulin: F_Diet_(1, 50) = 25.81, *p* < 0.0001, F_Treatment_(3, 50) = 4.721, *p* = 0.0056, F_Interaction_(3, 50) = 1.138, *p* = 0.3426; leptin: F_Diet_(1, 53) = 48.86, *p* < 0.0001, F_Treatment_(3, 53) = 5.908, *p* = 0.0015, F_Interaction_(3, 53) = 2.091, *p* = 0.1125; ghrelin: F_Diet_(1, 53) = 90.95, *p* < 0.0001, F_Treatment_(3, 53) = 8.422, *p* = 0.0001, F_Interaction_ (3, 53) = 0.6737, *p* = 0.5720). Within the STD condition, serum insulin levels were significantly higher in the 100 ng Mar1 group compared to the control group (*p* = 0.025), while no other significant differences were observed among STD-fed groups (Figure 2A). The corresponding comparison against the vehicle group was not significant (100 ng vs. vehicle, *p* = 0.9919), and serum insulin did not differ between the untreated control and vehicle groups (*p* = 0.0507). On the other hand, 50 and 100 ng Mar1 infusion significantly increased serum leptin levels in STD-fed animals compared with control animals (*p* = 0.0003 and *p* = 0.0091, respectively; Figure 2B), while only 50 ng Mar1 significantly elevated ghrelin compared with both control and vehicle groups in STD-fed animals (*p* = 0.0016 and *p* = 0.0055, respectively; Figure 2C) Within the HFD condition, no pairwise comparison between Mar1 and control or vehicle reached significance, with the closest being ghrelin in the 50 ng group versus vehicle (*p* = 0.051; Figure 2C).

**Figure 3:**
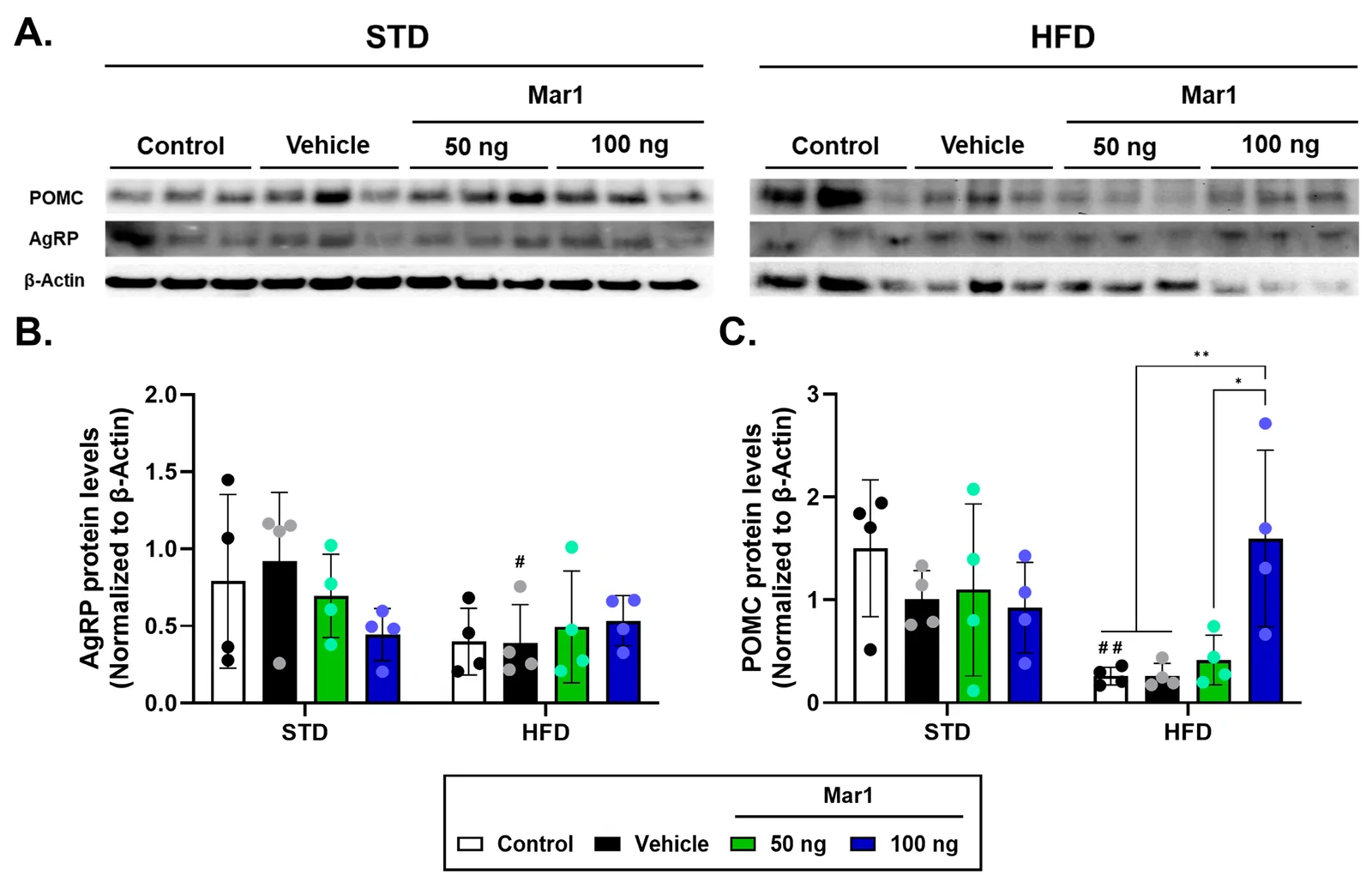
Effect of Mar1 infusion on POMC and AgRP protein levels in the ARC of animals fed either an STD or an HFD (**A**) Representative immunoblot images. Relative (**B**) AgRP and (**C**) POMC levels, normalized to β-actin, for both dietary conditions. (Statistical analysis: Two-way ANOVA followed by Tukey’s multiple comparison test. ANOVA followed by Tukey’s multiple comparison test. Asterisks denote comparisons between treatment groups within a diet (* *p* < 0.05, ** *p* < 0.01); hash symbols denote comparison of the same treatment group between diets (^#^ *p* < 0.05 and ^##^ *p* < 0.01 vs. respective treatment-matched STD-fed group).

Next, we investigated whether Mar1 infusion alters the AgRP and POMC levels in the hypothalamus. Infusion of Mar1 did not alter AgRP expression in the ARC (Figure 3A,B, Figures S1 and S2, Western blot: F_Diet_ (1, 24) = 4.857; *p* = 0.0374, F_Treatment_(3, 24) = 0.347, *p* = 0.7914, F_Interaction_(3,24) = 1.309, *p* = 0.2945). For POMC, by contrast, the diet x treatment interaction was significant and accounted for 27.9% of the total variation (F_Diet_ (1, 24) = 7.136; *p* = 0.0134, F_Treatment_(3,24) = 2.109, *p* = 0.1256, F_Interaction_(3, 24) = 4.826, *p* = 0.0091), indicating that the effect of Mar1 on POMC expression depended on the dietary condition. On the other hand, there were no significant differences in POMC protein levels between the vehicle and Mar1-treated animals under STD (Figure 3A,C, Figures S3 and S4), whereas under HFD 100 ng, Mar1 raised POMC above the control, vehicle and 50 ng groups (*p* = 0.0076, *p* = 0.0078 and *p* = 0.0204, respectively; Figure 3A,C, Figure S4).

## 3. Discussion

In this study, we demonstrated that ICV Mar1 infusion alters hypothalamic appetite-regulating neuropeptides and metabolic hormones in male rats. Notably, Mar1 administration increased POMC expression without significantly altering the AgRP levels in the ARC. This increase in POMC was confined to HFD-fed animals and was supported by a significant diet x treatment interaction. Mar1 also produced significant treatment effects on serum insulin, leptin, and ghrelin. However, none of the hormone endpoints showed a significant diet x treatment interaction. Because food intake and body weight were unchanged, these results demonstrate changes in measured neuroendocrine endpoints rather than an improvement in energy balance or obesity.

The high-fat feeding protocol produced the intended phenotype, although not for every measure. HFD-fed rats gained 17.3% more weight than STD-fed rats over the 12-week feeding period and showed a significant main effect of diet on all three circulating hormones, with leptin, the hormone most directly reflecting adipose mass, accounting for 38.1% of the total variance. The Lee index, by contrast, did not discriminate between the diets. The reason for this is apparent in our own measurements. Naso-anal length increased in parallel with body weight under HFD (24.6 ± 1.0 cm vs. 23.8 ± 1.0 cm, *p* = 0.003), so the ratio was preserved. This observation is consistent with previous reports that the Lee index correlates poorly with carcass fat in adult rats, because naso-anal length is a weak predictor of fat-free mass in animals of similar age and nutritional history [44,45]. Therefore, the Lee index was not used as a primary criterion for obesity confirmation in this study. The success of the dietary manipulation is therefore better judged from body weight gain (17.3% higher under HFD, *p* < 0.0001) and the expected endocrine profile, characterized by a significant main effect of diet on all three circulating hormones (all *p* < 0.0001). However, it should be acknowledged that direct measures of adiposity, such as epididymal or retroperitoneal fat pad weights or whole-body composition analysis, were not obtained. Such measurements would have provided stronger confirmation of the obese phenotype and are a limitation of the present study.

An important question is how the neuroendocrine changes should be interpreted given that neither dose of Mar1 altered body weight or food intake. The findings describe a shift in hypothalamic and circulating signaling rather than a demonstrated change in energy balance, for two reasons. First, seven days is short relative to the interval over which a change in ARC POMC expression would be expected to become visible as a difference in adiposity. POMC neuron stimulation reduces food intake only over a 24 h protocol, whereas AgRP activation drives feeding within minutes [46], and centrally or systemically administered pro-resolving mediators required 10 to 20 days to improve metabolic phenotypes in obese rodents [40,41,47]. Moreover, POMC is a prohormone requiring convertase cleavage and granule storage before α-MSH is released in a stimulus-dependent manner [48], so an increase in total POMC protein does not by itself establish increased α-MSH release or melanocortin receptor activation. Second, AgRP expression was unchanged, and because NPY/AgRP neurons make direct synaptic contact with POMC neurons and tonically inhibit them through NPY and GABA [49], an increase in POMC without a reciprocal fall in AgRP need not produce a net anorexigenic drive at downstream melanocortin receptors [22,50]. We therefore interpret the present findings as evidence that central Mar1 engages hypothalamic and endocrine signaling, and not as evidence that Mar1 alters energy homeostasis; establishing the latter would require longer infusions combined with direct measurement of energy expenditure, body composition and meal patterning.

Mar1 is a DHA-derived lipid mediator with potent anti-inflammatory and pro-resolving actions and has been shown to improve insulin sensitivity and attenuate obesity-associated inflammation in peripheral tissues [40,41,42,51]. Previously, ICV resolvin D2 (RvD2) administration was shown to reverse hypothalamic leptin resistance and reduce visceral adiposity in obese rodents [47]. Moreover, ICV omega-3 fatty acid administration was reported to improve leptin and insulin sensitivity [52]. In our study, central Mar1 increased POMC without a corresponding change in AgRP. A selective rise in POMC would be expected to favor anorexigenic tone, since POMC neurons release α-MSH to activate melanocortin receptors that suppress appetite, whereas AgRP neurons normally antagonize this effect [50].

One mechanism that could account for the effects of Mar1 on appetite-related neuropeptides and hormone profiles is its anti-inflammatory and pro-resolving action within the hypothalamus. This possibility is developed below from the existing literature, since no inflammatory, glial or inflammatory signaling marker was measured in the present design. Chronic HFD feeding activates glial cells in the ARC and provokes the release of pro-inflammatory cytokines [53], which engage IκB kinase-β (IKKβ)/NF-κB and c-Jun N-terminal kinase (JNK) signaling and upregulate suppressor of cytokine signaling 3 (SOCS3) in POMC and AgRP neurons [53]. This is a hallmark of diet-induced hypothalamic inflammation and induces leptin and insulin resistance at the cellular level [52,53]. Because leptin receptor signaling in POMC neurons normally proceeds through STAT3 to drive POMC transcription [52], elevated SOCS3 and NF-κB activity in an inflamed hypothalamus blunts this pathway and uncouples peripheral nutrient status from central neural responses [54]. Additionally, in the present study, serum leptin was higher in STD-fed rats receiving Mar1 than in untreated controls, although this difference did not persist when the same groups were compared with the vehicle group. Circulating leptin concentrations track adipose mass and, in obesity, rise alongside leptin resistance rather than alongside improved sensitivity; therefore, an elevated concentration is as compatible with reduced central responsiveness as with more efficient signaling [55,56]. Distinguishing the two requires functional evidence, such as a leptin challenge with assessment of downstream STAT3 activation or an accompanying change in food intake, and the present design provides neither.

Moreover, central Mar1 may also influence ghrelin responsiveness. Ghrelin is known to trigger hypothalamic AgRP/NPY release to stimulate appetite [57], and DIO causes ghrelin resistance in the AgRP/NPY neurons [58]. Resolving inflammation could modulate ghrelin receptor expression or signaling in AgRP and POMC neurons. The ghrelin response, however, occurred under STD rather than HFD conditions, so whether central Mar1 modifies orexigenic drive in diet-induced obesity remains to be determined. Interestingly, in STD-fed rats, the 50 ng Mar1 dose significantly increased circulating ghrelin without altering POMC expression. It is plausible that Mar1 may directly influence ghrelin secretion through peripheral mechanisms, such as via the vagal nerve or gastric pathways, independently of its direct effects on POMC neurons in the ARC. This hypothesis warrants further investigation into the potential gut–brain axis-mediated effects of central Mar1 administration. Notably, previous studies have demonstrated that Mar1 can modulate adipokine expression and secretion in both in vivo and in vitro models, supporting the concept that it exerts direct effects on endocrine cells beyond its central actions [59]. Furthermore, SPMs, including Mar1, are known to activate specific G-protein-coupled receptors and nuclear receptors such as RORα, which are expressed in peripheral tissues and may mediate gut-related effects [60]. Additionally, emerging evidence indicates that SPMs can modulate the function of peripheral immune cells and tissue-resident macrophages, which may indirectly influence endocrine signaling through the gut–brain axis [60,61].

The direction of these changes also merits comment, since a rise in circulating ghrelin, an orexigenic signal, together with an increase in ARC POMC, an anorexigenic precursor, protein levels is counterintuitive. The two need not represent a single coordinated response. The increase in ghrelin was confined to STD-fed animals at a lower dose and the POMC increase to HFD-fed animals at a higher dose, and only the latter was accompanied by a significant diet x treatment interaction. Where the two coincide, a counter-regulatory relationship is plausible, since ARC neurons modulate autonomic outflow to the gastrointestinal tract [50] and a rise in an orexigenic peripheral signal is the expected homeostatic answer to increased central melanocortin tone, which would also accord with the unchanged food intake we observed. Alternatively, the ghrelin signal may simply not have been transmitted. Ghrelin acts principally on ARC NPY/AgRP neurons, which become ghrelin-resistant in DIO [58], and AgRP expression was unchanged under both diets here. A rise in circulating ghrelin without a corresponding change in its principal hypothalamic target would leave melanocortin output unopposed. However, it must be emphasized that this counter-regulatory interpretation remains speculative, as we did not measure acyl-ghrelin (the bioactive form), growth hormone secretagogue receptor (GHSR) expression, or AgRP neuronal activity in the present study. Future studies should specifically test whether the ghrelin increase represents a homeostatic response to enhanced melanocortin tone or alternatively reflects impaired ghrelin sensitivity in the context of diet-induced obesity.

The formal evidence for diet dependence is confined to POMC protein abundance. The insulin and leptin responses under STD were significant against the untreated control group but not against the vehicle group (insulin, vehicle vs. 100 ng, *p* = 0.9919; leptin, vehicle vs. 50 ng, *p* = 0.0875 and vs. 100 ng, *p* = 0.6195), so a contribution of the surgical and infusion procedure to these two endpoints cannot be excluded; the ghrelin response under STD (vehicle vs. 50 ng, *p* = 0.0055) and the POMC response under HFD (vehicle vs. 100 ng, *p* = 0.0078) were significant against both comparators. In STD-fed animals, Mar1 shifted insulin, leptin and ghrelin in the same direction as high-fat feeding itself, although the concentrations reached under STD did not match those of untreated HFD-fed rats and the main effect of diet remained significant for all three hormones. This resemblance did not extend to ARC POMC, which was markedly lower under HFD and was not significantly altered by Mar1 under STD. Central Mar1 exposure in metabolically healthy animals therefore cannot be assumed to be metabolically favorable. For insulin, leptin, and ghrelin, the non-significant interactions mean that significance of simple comparisons only within STD cannot be taken as evidence that Mar1 acts differently between diets. Elevated and more variable HFD baselines may have reduced the ability to resolve individual pairwise contrasts, particularly since diet-induced obesity reduces the responsiveness of arcuate neurons to leptin, insulin and ghrelin, but this is a descriptive possibility rather than a demonstrated mechanism [20,52,53,58]. Accordingly, the central POMC and peripheral hormone findings are interpreted as parallel observations, not as proof of a brain–periphery pathway or restored hypothalamic hormone sensitivity. An increase in POMC without a parallel change in circulating hormones would be consistent with central effects of Mar1 on hypothalamic neuropeptides occurring independently of changes in peripheral hormone concentrations, possibly by enhancing neuronal sensitivity to these hormones within the central nervous system. Such a mechanism would be consistent with the pro-resolving pharmacology of Mar1, but was not addressed by the present endpoints. This interpretation aligns with the established role of SPMs in resolving inflammation and improving metabolic homeostasis through both peripheral and central mechanisms [33,59], and with evidence that Mar1 can regulate tissue-specific expression of metabolic mediators without necessarily altering circulating hormone levels [59]. Notably, recent reviews have highlighted that SPMs exert their effects in the central nervous system through multiple mechanisms, including modulation of glial cell function, regulation of neuroinflammatory pathways, and restoration of tissue homeostasis [61,62]. Whether Mar1 enhances hypothalamic sensitivity to peripheral metabolic signals by resolving obesity-associated neuroinflammation and restoring glial-neuronal crosstalk remains to be established.

As an SPM, Mar1 actively drives the resolution of inflammation, a process distinct from mere anti-inflammatory suppression [63]. Rather than broadly inhibiting immune function, Mar1 engages specific pathways that shift immune cells from a pro-inflammatory state to a pro-resolving, reparative state [40]. Within the hypothalamus, it can be suggested that Mar1 dampens microglial activation and cytokine release. This could occur through known actions of Mar1 on macrophage-lineage cells, as studies have shown that Mar1 stimulates a phenotypic switch from classically activated (M1) to alternatively activated (M2) macrophages [64]. In our study, however, no marker of neuroinflammation, glial activation or inflammatory signaling was measured, and leptin and insulin sensitivity were not assessed functionally. Whether Mar1 resolves hypothalamic inflammation and thereby resensitizes ARC neurons to peripheral hormonal cues therefore remains an untested hypothesis in the present work. Leptin, which is already present at high levels in HFD rats, could in principle activate POMC neurons once more, as suggested by the rise in POMC expression [53].

Beyond the general anti-inflammatory and pro-resolving actions, Mar1 might also exert more direct, receptor-mediated effects on neural cells. Many SPMs signal through G-protein-coupled receptors on target cells. A relevant example is the receptor for RvD2, GPR18, which, as reported, is present on POMC and NPY/AgRP neurons [47]. While the specific cognate receptor for Mar1 in the brain is not yet fully identified, recent evidence from adipose tissue research suggests that leucine-rich repeat-containing G-protein-coupled receptor 6 (LGR6) is a Mar1 receptor in peripheral cells [65]. Moreover, another target of Mar1, RORα, is expressed in various regions in the nervous system, including the hypothalamus [66]. By modulating the activities of its receptor, Mar1 may directly regulate the POMC and AgRP neuron activity. It is also plausible that Mar1 receptors on microglia transduce signals that result in the release of neurotrophic or anti-inflammatory factors benefiting neurons. While speculative, these direct actions could complement the broad anti-inflammatory milieu to rapidly adjust neuronal activity. In the case of RvD2, central administration was reported to upregulate hypothalamic anti-inflammatory mediators [47]. Under HFD conditions, not only are POMC neurons less active, but their structural and synaptic inputs are altered; studies have found reduced excitatory input and enhanced inhibitory input onto POMC cells in obese rodents, partly due to inflammatory processes, whereas AgRP neurons show the opposite trend [67]. By resolving inflammation, Mar1 may allow a reversal of these synaptic changes. Although we did not examine synaptic markers, it can be speculated that Mar1 could increase excitatory drive to POMC neurons or the intrinsic excitability of these cells, while dampening the overactive AgRP network.

Our study had several limitations. Most importantly, no inflammatory readout was included in the present design. We did not measure expression of hypothalamic cytokines such as *Tnfa*, *Il1b* or *Il6*, markers of glial activation such as GFAP or Iba1, or components of inflammatory signaling such as SOCS3 or phosphorylated NF-κB p65. The pro-resolving mechanism outlined above is therefore inferred from the established pharmacology of Mar1 and from prior studies of central SPM administration and remains to be tested directly. Second, the Mar1 doses were adopted from a study in which they were given systemically to mice in a model of acute liver injury [68] and were transposed here to continuous central infusion in rats. Although the resulting estimated cerebrospinal fluid (CSF) concentrations fall within the range over which Mar1 is bioactive in vitro, the doses remain pharmacological rather than physiological. Endogenous CSF Mar1 concentrations in the rat have not been established; therefore, the extent to which our infusions reproduce a physiologically attainable central Mar1 tone is unknown. Additionally, direct measures of adiposity, such as fat pad weights or body composition analysis, would have strengthened confirmation of the obesity phenotype beyond body weight gain and the hormone profile, and no glucose tolerance or insulin sensitivity test was performed. Moreover, because immunoblotting was performed on whole arcuate nucleus lysates, the measured POMC and AgRP signals cannot be resolved to individual cells. The ICV infusion was relatively short-term, so we cannot infer chronic effects on food intake or body weight. Moreover, we focused on food intake and levels of neuropeptides and hormones, but additional pathways, including autophagy and endoplasmic reticulum stress, may mediate central actions of Mar1. Detailed electrophysiological or receptor-binding studies would also clarify whether Mar1 directly modulates leptin, insulin, or ghrelin receptor signaling in ARC neurons.

## 4. Materials and Methods

### 4.1. Study Design and Animal Husbandry

**Figure 4:**
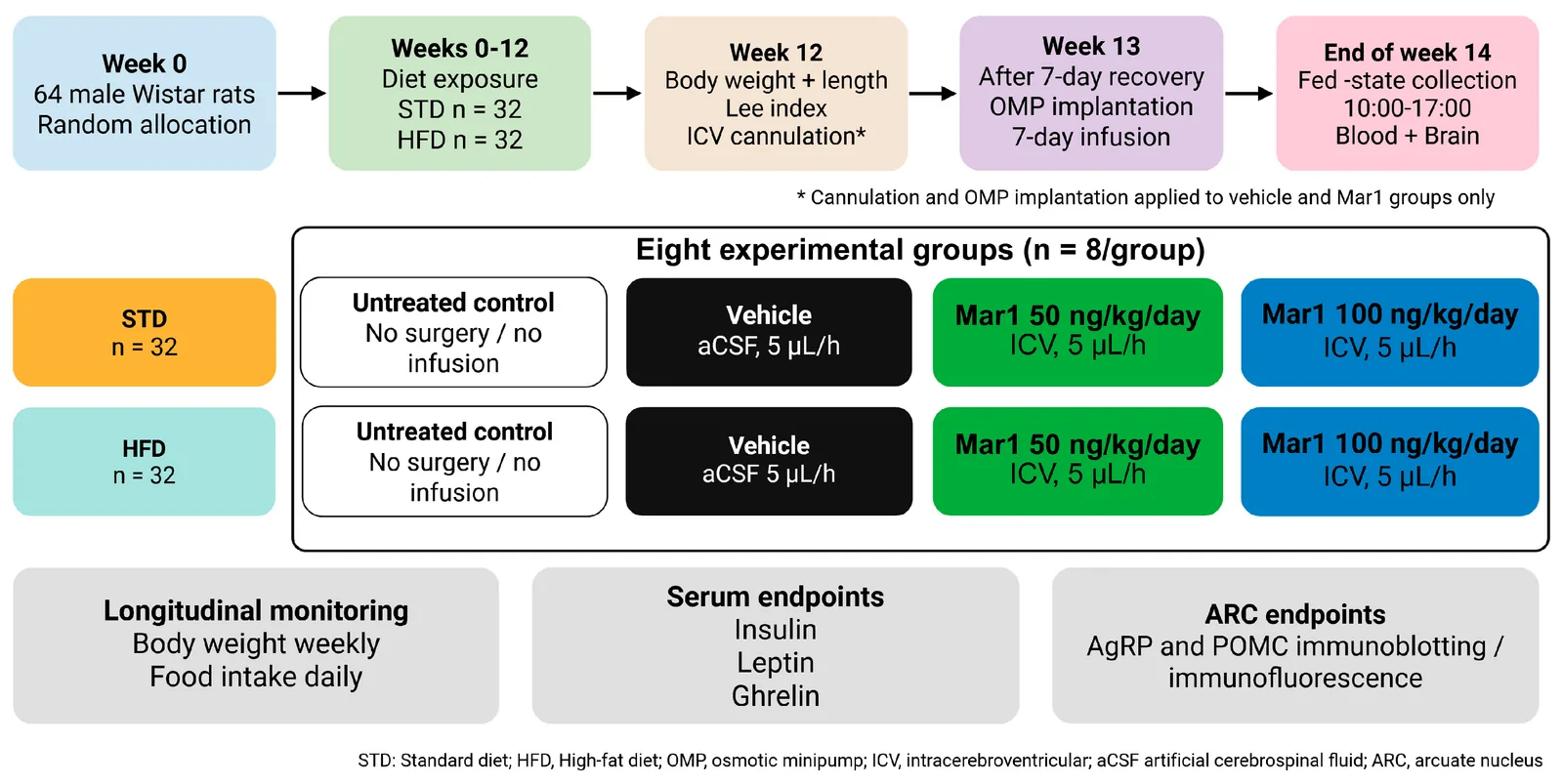
Schematic of the experimental design. Male Wistar albino rats (*n* = 64) were fed either a standard diet (STD) or a high-fat diet (HFD, 60% fat) ad libitum for 12 weeks, at which point body weight, naso-anal length and the Lee index were recorded. Within each dietary condition, animals were randomized to one of four arms (*n* = 8 per group). Groups included an untreated control that underwent neither surgery nor infusion, a vehicle group receiving artificial cerebrospinal fluid, and two Mar1 groups receiving 50 or 100 ng/kg/day. An intracerebroventricular cannula was implanted in the lateral ventricle (AP = −0.8 mm, ML = 1.4 mm, DV = 4.8 mm) and, after a recovery period, connected to a subcutaneously implanted osmotic minipump (ALZET 2ML2, Campbell, CA, USA), delivering 5 µL/h for 7 days. All animals were sacrificed on day 7 of infusion, on the same day, between 10:00 and 17:00 during the light phase, in a fed state and in randomized order with respect to group. Serum was collected for insulin, leptin and ghrelin, and arcuate nucleus POMC and AgRP were assessed by immunoblotting and immunofluorescence.

All experimental procedures were conducted in accordance with the institutional and national ethical guidelines for the care and use of laboratory animals (Approval no: 2024/7-1). All animals were housed at the Inonu University Experimental Animals Research and Production Center (INU-DEHUM, Malatya, Türkiye) under controlled environmental conditions (21 ± 1 °C; 12 h light/dark cycle) with ad libitum access to tap water and assigned diet [69]. The sample size was calculated using power analysis with a type I error (α) of 0.05, a type II error (β) of 0.20 (Power = 0.80), a maximum predicted difference between groups of 0.6, a standard deviation of 0.2, and a minimum of eight rats per group with a total of 8 groups. To minimize subjective bias, authors who conducted the experiments and/or performed the statistical analyses were blinded to the experimental groups. A total of 64 male Wistar albino rats weighing 48–61 g (mean 55.2 ± 3.8 g) were randomly assigned to eight groups with eight animals and were fed ad libitum either with an STD or an HFD (20% carbohydrate, 20% protein, and 60% fat) for 12 weeks (*n* = 32 animals for each feeding regimen; Figure 4). At week 12, obesity was assessed in HFD-fed rats by utilizing the Lee index, which is calculated by dividing the cube root of body weight (in grams) by the naso-anal length (in centimeters), and animals with a Lee index > 0.3 were classified as obese (Figure 4). The index did not discriminate between the two dietary conditions, and high-fat diet feeding was therefore verified from body weight gain and the circulating hormone profile [45].

Following the 12-week feeding period, rats within each diet condition were assigned to one of four treatment arms (*n* = 8 per group), yielding eight total experimental groups as follows: control, vehicle, 50 ng/kg/day Mar1 and 100 ng/kg/day Mar1 (Figure 4). Within each diet condition, control groups served as an untreated diet-only control group with no surgery or infusion procedures, while other groups received intracerebroventricular (ICV) infusion of artificial CSF (aCSF; 126 mM NaCl, 2.5 mM KCl, 2.4 mM CaCl2, 1.2 mM MgCl2, 11 mM D-glucose, 1.4 mM NaH2PO4, and 25 mM NaHCO3; pH = 7.4) as the vehicle, or 50 ng/kg/day or 100 ng/kg/day Mar1 (10878, Cayman Chemical, Ann Arbor, MI, USA) dissolved in aCSF for one week via osmotic minipumps (OMPs). The doses of 50 and 100 ng/kg/day were adopted from Zhang et al., in which both attenuated hepatic injury and inflammatory signaling were considered in mice, with a higher dose producing a more significant effect [68]. The two-fold separation was retained in order to test for dose-dependence. Those doses were administered systemically to mice, so their transposition to continuous ICV infusion in rats could be understood as ensuring centrally active exposure rather than reproducing the exposure achieved in the original study. Pumps were loaded individually according to body weight (mean 418 ± 29 g under STD and 478 ± 39 g under HFD), delivering approximately 21–24 ng/day at the lower dose and 42–48 ng/day at the higher dose. Assuming a rat CSF formation rate of 1.8–2.8 µL/min [70], the estimated steady-state CSF concentration was approximately 5–18 ng/mL (14–51 nM), close to the 10–50 nM at which Mar1 is active in vitro [68] and above the 0.01–10 nM range for LGR6 activation [37], which is an appropriate margin given the poor parenchymal penetration of ventricularly delivered compounds [71]. Food intake was recorded daily for each animal, and body weight was measured weekly throughout the 14-week experimental period (Figure 4).

### 4.2. Stereotaxic Implantation of Brain Infusion Cannula and Osmotic Minipumps

Rats were anesthetized by intraperitoneal injection of ketamine (70 mg/kg) and xylazine (8 mg/kg) and the brain infusion cannula was inserted into the lateral ventricle (AP = −0.8 mm and ML = 1.4 mm relative to the sagittal suture and DV = 4.8 mm from the skull surface). The cannula was secured to the skull using dental cement; the incision was sutured; and the wound area was treated with 10% povidone–iodine. After a 7-day recovery period, animals were re-anesthetized and the osmotic minipump was connected to the cranial cannula tubing. The connected pump was implanted subcutaneously, and continuous ICV infusion was maintained for 7 days at 5 µL/h (ALZET; model 2ML2; 2 mL reservoir; nominal duration 14 days). The selected rate was the nominal delivery rate of the ALZET 2ML2 pump, which is directly supported by chronic lateral-ventricular infusion studies in adult rats that used 5 µL/h for 14 days [72,73,74]. The pump delivered approximately 120 µL/day and 840 µL over the entire 7-day infusion period. Although this volume appears large in absolute terms, it is modest relative to CSF dynamics at a rat CSF formation rate of 1.8–2.8 µL/min (approximately 2.6–4.0 mL/day); the infused volume corresponds to 3.0–4.6% of the daily CSF production [70]. Thus, 5 µL/h is within established chronic ICV practice in adult rats and is small relative to continuous CSF formation and turnover. Because infusion rate and volume can influence ventricular distribution, the vehicle and both Mar1 groups received the identical 5 µL/h rate; the vehicle group therefore controlled for effects attributable to the infusion procedure and aCSF delivery.

### 4.3. Blood and Brain Tissue Collection

At the end of the 7-day ICV infusion period, rats were deeply anesthetized and whole blood was obtained by terminal cardiac puncture. All animals were killed on the same day between 10:00 and 17:00, that is, during the light (inactive) phase, and food was available *ad libitum* until the moment of anesthesia. Collection during the light phase was chosen because rats consume most of their food during the dark phase, so this window minimizes acute postprandial variation in circulating hormones, ghrelin in particular. Animals were processed in randomized order across experimental groups, so that each group was distributed across the collection window and treatment was not confounded with time of day. Animal processing and tissue collection were performed in parallel to keep the interval between anesthesia and tissue freezing short and uniform. Serum was separated and stored at −80 °C until hormone and biochemical assays were performed. For Western blot analyses, animals were perfused with cold 0.9% NaCl, the brain was dissected, snap-frozen on dry ice and stored at −80 °C. For immunofluorescence analyses, animals were perfused with 4% paraformaldehyde (PFA) in phosphate-buffered saline, and brain tissue was dissected and fixed in 4% PFA overnight at 4 °C. Fixed brains were submerged in 30% sucrose in PBS to cryoprotect, embedded in OCT and stored at −80 °C prior to sectioning.

### 4.4. Serum Hormone Levels

Serum samples were thawed on ice, and serum leptin (Coon Koon Biotech, Shangai, China, CK-bio, 14910), ghrelin (Coon Koon Biotech, CK-bio, 14676), and insulin (Coon Koon Biotech, CK-bio, 14821) concentrations were measured using enzyme-linked immunosorbent assay (ELISA) according to the manufacturers’ instructions.

### 4.5. Immunoblotting

The ARC was dissected from the frozen tissues and homogenized in RIPA lysis buffer (Santa Cruz Biotechnology, Dallas, TX, USA, sc-24948) supplemented with a protease and phosphatase inhibitor mix (Halt™ Protease and Phosphatase Inhibitor Cocktail, Thermo Scientific™, Waltham, MA, USA) using a Bullet Blender X24 (NextAdvance, Troy, NY, USA). Total protein concentration in the lysates was determined using the BCA protein assay kit (Pierce™ BCA Protein Assay Kit, Thermo Scientific™) and samples were stored at −80 °C until use. Equal amounts of total protein lysates (30–40 µg per lane) were resolved using 4–12% Bis-Tris Gels (Bolt™ 4–12% Bis-Tris Plus Gels, Invitrogen, Waltham, MA, USA) and transferred to 0.45 μm PVDF membranes (Thermo Scientific™) using semi-dry transfer with the Towbin transfer buffer (25 mM Tris, 192 mM Glycine, 20% methanol) at 22 V for 30 min at room temperature. The membrane was blocked in 5% (*w*/*v*) skim milk prepared in Tris-buffered saline containing 0.1% Tween-20 (TBS-T) for 1 h at room temperature and incubated with rabbit anti-AgRP (1:500, Abcam, Cambridge, UK, ab113481, RRID: AB_10862532 [75]) and rabbit anti-POMC (1:1000, Abcam, ab254257, RRID: AB_3102024) antibodies at 4 °C overnight. After washing with TBS-T, the membrane was probed with an HRP-conjugated anti-rabbit antibody (1:2000, Cell Signaling Technology, Danvers, MA, USA, 7074, RRID: AB_2099233). The membrane was stripped using a Western BLoT Stripping Buffer (Takara Bio, San Jose, CA, USA T7135A) prior to further rounds of incubation. β-Actin (Direct-Blot™ HRP anti-β-actin Antibody, BioLegend, San Diego, CA, USA, 643807, RRID: AB_2566701) was used as the endogenous loading control for normalization of protein expression. Immunoblot images were acquired using a ChemiDoc XRS+ (Bio-Rad, Hercules, CA, USA) imaging system and bands were quantified using Image Lab™ software (version 6.0.0, Bio-Rad).

### 4.6. Confocal Microscopy

OCT-embedded brain tissues were transferred to −20 °C and coronal sections were cut on a cryostat. Sections were advanced until atlas-based anatomical landmarks were visible (lateral ventricle, third ventricle, corpus callosum, and optic chiasm) to localize the ARC. In total, 10 µm thick coronal sections were mounted onto positively charged slides and dried at 37 °C. Sections were washed three times with PBS for 10 min, incubated in PBS with 0.5 M glycine for 45 min at room temperature and permeabilized for 30 min in 0.1% Triton X-100 in PBS at 4 °C. Sections were then blocked in PBS containing 5% bovine serum albumin (BSA) and 0.05% Triton X-100 at room temperature for 45 min. Primary antibodies against AgRP (4 μg/mL, R&D Systems, Minneapolis, MN, USA, AF634) and POMC (4 μg/mL, Novus Biologicals, Centennial, CO, USA, NB100-1533) were prepared in PBS with 1% BSA and 0.05% Triton X-100 and applied to the tissue sections for overnight incubation at 4 °C. Following incubation, sections were washed three times with PBS for 10 min and incubated with an Alexa Fluor^®^ 594-conjugated secondary antibody (1:2000, Abcam, ab150132) for 1 h at room temperature. After three further washes with PBS for 10 min, slides were then coverslipped with a mounting medium (Fluoroshield ™ with DAPI; Sigma, Saint Louis, MO, USA, F6057). Confocal imaging was performed using a confocal microscope (Zeiss, LSM700, Axio Imager 2) equipped with appropriate lasers (Laser wavelengths: 488 nm; 2.00%, 555 nm; 2.00% and 405 nm; 2.00%) and filters (Filters: 585–100, 420–480) at 10× (objective: EC Plan-Neofluar 10×/0.30 M27) and 20× (objective: Plan-Apochromat 20×/0.8 M27) magnifications under a controlled environmental temperature of 18.0 ± 2.0 °C. Imaging was performed using ZEN Blue 3.10 (Zeiss, Jena, Germany). AgRP and POMC immunoreactivity in ARC was quantified using ImageJ (version 1.54p) by an investigator blinded to the experimental groups [76,77]. For each animal (*n* = 3 per group), the mean fluorescence intensity was measured, background fluorescence was subtracted and data were normalized to the mean fluorescence intensity of control group measurements.

### 4.7. Statistical Analysis

Statistical analyses were conducted using GraphPad Prism^®^ 10.1 (GraphPad Software, Boston， MA, USA). The authors performed the experiments, and the statistical calculations were blinded to minimize bias. Data were expressed as mean ± standard deviation. Homogeneity of variances was assessed using Levene’s test. The distribution of data was assessed using the Shapiro–Wilk test. Data were analyzed by two-way ANOVA with diet (STD and HFD) and treatment (control, vehicle, 50 ng Mar1, and 100 ng Mar1) as between-subject factors, and the diet x treatment interaction was evaluated in every case. Pairwise comparisons were made using Tukey’s test. Body weight and food intake were analyzed separately within each diet, with week and treatment as factors, by two-way ANOVA, except for food intake under HFD, where missing values required a mixed-effects (REML) model; pairwise comparisons were made using Tukey’s test. *p*-values lower than 0.05 were considered statistically significant.

## 5. Conclusions

The present findings indicate that 7-day central Mar1 infusion exerts a diet-dependent effect on hypothalamic melanocortin signaling, increasing arcuate nucleus POMC protein without a parallel change in AgRP levels under HFD. Mar1 also produced the main treatment effects on circulating insulin, leptin, and ghrelin, but these endocrine effects were not demonstrably diet-dependent and did not translate into changes in food intake or body weight. The findings therefore identify short-term neuroendocrine responses to pharmacological central Mar1 exposure; they do not establish enhanced hypothalamic hormone sensitivity, resolution of neuroinflammation, improvement in energy homeostasis, or therapeutic efficacy in obesity. Longer and mechanistically targeted studies are required to determine whether the POMC protein response has functional consequences and whether central Mar1 can be used safely and selectively in metabolic disease.

## Data Availability

The data that support the findings of this study are available from the corresponding authors upon reasonable request.

## Supplementary Materials

The following supporting information can be downloaded at https://www.mdpi.com/article/10.3390/ijms27188039/s1.

## Abbreviations

The following abbreviations are used in this manuscript:

aCSF	Artificial cerebrospinal fluid
AgRP	Agouti-related peptide
ANOVA	Analysis of variance
ARC	Arcuate nucleus
BSA	Bovine serum albumin
CSF	Cerebrospinal fluid
DHA	Docosahexaenoic acid
DIO	Diet-induced obesity
ELISA	Enzyme-linked immunosorbent assay
GFAP	Glial fibrillary acidic protein
GHSR	Growth hormone secretagogue receptor
HFD	High-fat diet
Iba1	Ionized calcium-binding adapter molecule 1
ICV	Intracerebroventricular
IKKβ	Iκb kinase-β
INU-DEHUM	Inonu University Experimental Animals Research and Production Center
JNK	C-Jun N-terminal kinase
LGR6	Leucine-rich repeat-containing G-protein-coupled receptor 6
M1	Classically activated macrophage
M2	Alternatively activated macrophage
Mar1	Maresin-1
NF-κB	Nuclear factor kappa-light-chain-enhancer of activated B cells
NPY	Neuropeptide Y
OMPs	Osmotic minipumps
PFA	Paraformaldehyde
POMC	Proopiomelanocortin
RORα	Retinoic acid-related orphan receptor alpha
RvD2	Resolvin D2
SOCS3	Suppressor of cytokine signaling 3
SPMs	Specialized pro-resolving mediators
STAT3	Signal transducer and activator of transcription 3
STD	Standard diet
T2D	Type 2 diabetes
TBS-T	Tris-buffered saline containing 0.1% Tween-20
TUBITAK	Scientific and Technological Research Council of Türkiye
α-MSH	α-Melanocyte-stimulating hormone
