# Supplementary material for: Diet-Dependent Effects of Intracerebroventricular Maresin-1 on Hypothalamus of Rats
Source: Int J Mol Sci. 2026 Sep 9;27(18):8039. doi: 10.3390/ijms27188039 (PMC13606587; doi:10.3390/ijms27188039)
Supplement: Supplementary file 1 [file ijms-27-08039-s001.zip › Supplementary Figure Captions.pdf]

Supplementary Figure Legends

Figure S1: AgRP immunoreactivity in the ARC of the animals fed with an STD. (A-C) Control, (D-F) vehicle, (G-I) 50 ng Mar1 and (J-L) 100 ng Mar1 groups (Blue color depicts DAPI while green color depicts AgRP). (M) Relative AgRP immunoreactivity.

Figure S2: AgRP immunoreactivity in the ARC of the animals fed with an HFD. (A-C) Control, (D-F) vehicle, (G-I) 50 ng Mar1 and (J-L) 100 ng Mar1 groups (Blue color depicts DAPI while green color depicts AgRP). (M) Relative AgRP immunoreactivity.

Figure S3: POMC immunoreactivity in the ARC of the animals fed with an STD. (A-C) Control, (D-F) vehicle, (G-I) 50 ng Mar1 and (J-L) 100 ng Mar1 groups (Blue color depicts DAPI while red color depicts POMC). (M) Relative POMC immunoreactivity.

Figure S4: POMC immunoreactivity in the ARC of the animals fed with an HFD. (A-C) Control, (D-F) vehicle, (G-I) 50 ng Mar1 and (J-L) 100 ng Mar1 groups (Blue color depicts DAPI while red color depicts POMC). (M) Relative POMC immunoreactivity.
